# Fully integrated electrically driven optical frequency comb at communication wavelength

**DOI:** 10.1515/nanoph-2022-0146

**Published:** 2022-06-03

**Authors:** Nanxi Li, Guanyu Chen, Leh Woon Lim, Chong Pei Ho, Jin Xue, Yuan Hsing Fu, Lennon Y. T. Lee

**Affiliations:** Institute of Microelectronics, A^*^STAR (Agency for Science, Technology and Research), 2 Fusionopolis Way, Singapore 138634, Singapore

**Keywords:** device, frequency comb, integrated photonics, laser, nanophotonics

## Abstract

To meet the high demand of data transmission capacity, optical communications systems have been developed. In order to increase the channel numbers for larger communication bandwidth, multi-mode lasers and laser arrays can be used. As an alternative coherent light source, optical frequency comb (OFC) contains multi-wavelength signal, and hence enables communication with data stream of tens of terabit/s. Fully integrated electrically driven OFCs are expected as a compact, robust, and low-cost light source for data communication. In this review article, the recent development progress on fully integrated electrically driven OFC generators are reviewed, with focus on the demonstrations in the past five years. Based on comb generation approaches, the works are categorized into two main types: one is OFC generators based on four-wave mixing in high-*Q* resonator, and the other is OFC generators based on mode-locked laser. At the end, a summary and future outlook are provided.

## 1 Introduction

Since the development of lightwave technology from 1970s, the bit-rate-distance product of a communication link has increased significantly to meet the high demand in communication speed [1]. Nowadays, a lot of activities in daily life including online shopping, interactive videos, tele-medicine, and cloud computing are enabled by high-speed optical communication systems. Within an optical communication link, lasers are used as coherent light sources for data transmission within the communication spectrum window. Furthermore, in the past two decades, integrated photonics technology based on silicon (Si) has been developed to make compact, robust, low power and low cost devices. The low cost of Si photonics technology is contributed by its complementary metal-oxide-semiconductor (CMOS)-compatible fabrication process. On Si photonics platform, functional devices including modulators [2], [3], [4], [5], tunable filters [6], [7], [8], [9], mode couplers [10], [11], [12], [13], photodetectors [14], [15], [16], [17] and nonlinear optical generators [18], [19], [20], [21] have been demonstrated. In addition, integrated lasers on Si substrate have been recently developed by using different approaches [22], [23], [24], [25], [26], [27], [28], [29] in order to make the system even more compact and robust. To further increase the number of communication channels, multimode lasers or laser arrays can be used [30], [31], [32], [33], [34], [35].

As alternative to multi-mode lasers or laser array as light sources, optical frequency comb (OFC) generates multi-wavelength coherent optical signals, and hence can be used as the source for high-capacity communication systems. OFC has been widely applied in different areas including microwave photonics [36], [37], [38], spectroscopy [39, 40], optical frequency synthesis [41], [42], [43] and distance ranging [44], [45], [46]. The nonlinear optical property of integrated photonics material (e.g., Si_3_N_4_) enables OFC generation on integrated photonics platform as well. The multispectral signal from chip-scale OFC can be used in communication systems and photonic interconnect in data centers [47], [48], [49], [50], [51], [52]. In 2017, the study by Palomo et al. [47] demonstrated soliton-based OFC for coherent optical communication. By using the multi-wavelength generated through four-wave mixing (FWM) within Si_3_N_4_ microring resonator, a data stream of >50 terabit/s on 179 optical carriers across the entire C and L bands are achieved. Although the microring resonators can be monolithically integrated on Si substrate for comb generation, the pump source reported in refs. [47], [48], [49] are off-chip. In order to make compact solid-state OFC sources, fully integrated electrically driven OFC generator is required. Since 2018, the electrically driven OFC generators formed by pump source integrated with high-*Q* resonator have been reported [52], [53], [54], [55], [56], [57], [58]. Remarkably, the heterogeneous laser integration approach reported in ref. [52] provides a pathway for the mass-production of the fully integrated OFC generator on wafer scale.

**Figure 1:**
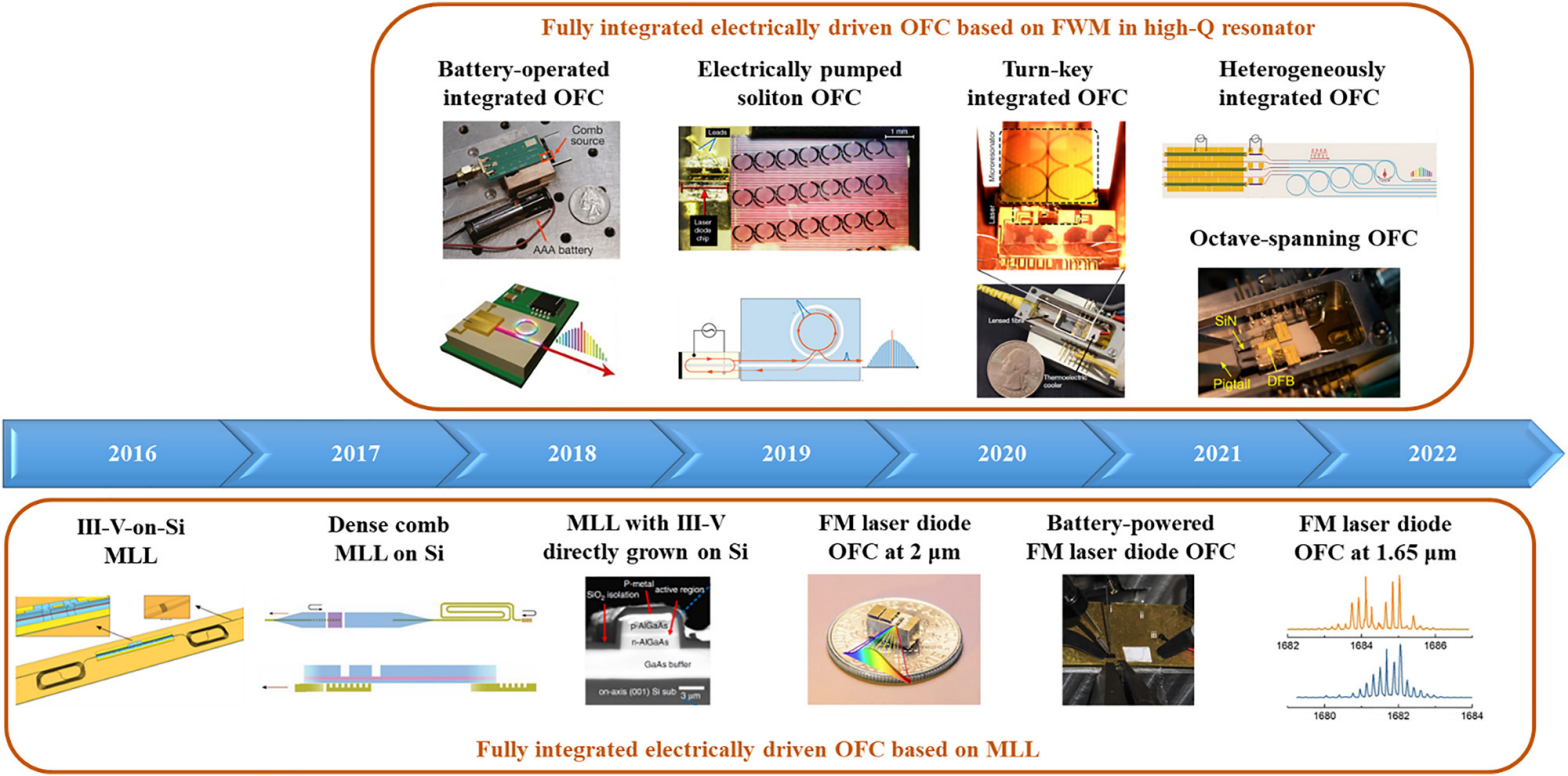
Research progress timeline for fully integrated electrically driven OFC at communication wavelength. (Top panel) **Battery-operated integrated OFC:** figures are adapted with permission from Springer Nature, Nature [53]. Battery-operated integrated frequency comb generator, by Stern et al. Copyright 2018. **Electrically pumped soliton OFC**: figures are adapted with permission from [54]. Licensed under a Creative Commons Attribution 4.0 International License. **Turn-key integrated OFC**: figures are adapted with permission from Springer Nature, Nature [55]. Integrated turnkey soliton microcombs, by Shen et al. Copyright 2020. **Heterogeneously integrated OFC**: figure is adapted from [52]. Reprinted with permission from AAAS. **Octave-spanning OFC**: figure is adapted from [56]. Licensed under a Creative Commons Attribution 4.0 International License. (Bottom panel) **III–V-on-Si MLL**: figure is adapted from [61] © The Optical Society. **Dense comb MLL on Si**: figure is adapted with permission from [63]. Licensed under a Creative Commons Attribution 4.0 International License. **MLL with III–V directly grown on Si**: figure is adapted with permission from [65]. © 2018 IEEE. **FM laser diode OFC at 2 μm**: figure is adapted with the permission from [69]. Licensed under a Creative Commons Attribution 4.0 International License. **Battery-powered FM laser diode OFC**: figure is adapted with the permission from [68]. Licensed under a Creative Commons Attribution 4.0 International License. **FM laser diode OFC at 1.65 μm**: figure is adapted with the permission from [70] © The Optical Society.

In addition to the integration of laser with high-*Q* resonator, the other major approach to make fully integrated electrically driven OFC generator is through mode-locked lasers (MLL). In the past five years, there are also a lot of research works demonstrating OFC generation based on MLLs [59], [60], [61], [62], [63], [64], [65], [66], [67], [68], [69], [70]. In this review, we have summarized the recent development progress of fully integrated electrically driven OFC generator, with the focus on the works reported in the past five years. The research progress timeline is shown in Figure 1. Section 2 is on OFC generation through FWM in high-*Q* resonator. Section 3 is on OFC generation through mode locking (ML). At last, Section 4 provides a summary and future outlook. For Sections 2 and 3, a table summarizing the key specifications of OFC is presented in each section. The key specifications in Tables 1 and 2 include materials, nanostructures, comb generation mechanisms, repetition rate, wavelength range, output power, and comb linewidth. The key results are selected and discussed in these two sections as well. Differentiating from recent comprehensive reviews on integrated OFC [71], [72], [73], [74], this review focuses the discussion on fully integrated electrically driven OFC demonstrated at telecommunication wavelength regimes, with recently reported key results presented in more detail.

## 2 OFC generation through FWM in high-*Q* resonator

As mentioned in the earlier section, key specifications of OFC including materials, nanostructures, comb generation mechanisms, repetition rate, wavelength range, output power, and comb linewidth are selected and summarized in Tables 1 and 2. Repetition rate and wavelength range are two key parameters in OFC design [73]. Lower repetition rate is generally preferred for the following two aspects. One aspect is that low repetition rates of tens of GHz or below allows for ease of access and compatibility to electronics for signal modulation in an optical communication system. The other aspect is that lower repetition rate gives higher spectrum resolution for spectroscopy application. In addition to lower repetition rate, a wide wavelength coverage in the communication windows (including O-band, S-band, C-band, and L-band) is expected as it means more channels and wider bandwidth (BW) for data transmission. Also, wide wavelength coverage enables more spectral information in spectroscopy. In addition, in high precision metrology (e.g., optical signal synthesis [41], [42], [43]), octave spanning is required for self-referencing purpose [75]. In the meanwhile, due to the total pump power constrain, it is hard to achieve both low repetition rate and wide wavelength range simultaneously. The trade-off needs to be balanced in OFC generation for specific application. A map of repetition rate and wavelength coverage for current integrated OFC technologies can be found in ref. [73]. Besides repetition rate and wavelength range, output power and comb linewidth of OFC are also important parameters. High output power (on mW level) is expected for transmission in a communication system due to the power budget within the communication link. Higher optical power enables better system-level performance in terms of higher signal-to-noise ratio and lower bit error rate, which are necessary for an optical communication system. At the same time, optical amplifiers can be used after the source to boost up the optical power in communication links. Also, narrow comb linewidth at the level of kHz or lower is expected in a high-performance optical communication system as narrow linewidth enables longer transmission distance and higher tolerance for complex information modulations.

From Table 1, it can be found that OFCs based on FWM in high-*Q* resonator typically can achieve repetition rate from tens of GHz up to 1 THz. The wavelength range covers tens of nm up to 1 μm (one octave) [56], which is generally wider than the MLL-based OFC (as summarized in Table 2). The FWM-based OFC total output power can be up to mW level but the power per comb line is typically in sub-mW scale. Such output power is limited by the optical conversion efficiency of the FWM process, which is typically below 5% [44, 53, 73, 76]. The comb linewidth in FWM-based OFCs is typically on sub-kHz level and can even reach a few hertz [58], which is narrower than the comb linewidth obtained by MLL approach (as summarized in Table 2). Such narrow comb linewidth is mainly contributed by the self-injection locking (SIL) of pump laser diode with microresonator. More details on SIL will be discussed in later part of current section.

**Table 1:** Summary of fully integrated electrically driven OFC based on FWM in high-*Q* resonator.

Materials and	Comb generation	Resonator intrinsic	Repetition	Wavelength	Output	Comb	Remarks	Reference/
structures	mechanism	cavity *Q*	rate	range/span	power	linewidth		year
Si_3_N_4_ microring resonator	FWM	(8.0 ± 0.8) × 10^6^	194 GHz	1530–1630 nm	0.24 mW (total comb power)	40 kHz	III–V amplifier edge-coupled to Si_3_N_4_ chip; OFC generation system is battery operated	[53]/2018
Si_3_N_4_ microring resonator	FWM	>1 × 10^7^	149 and <100 GHz	1500–1560 nm	N.A.	∼201 kHz	III–V laser diode butt coupled to Si_3_N_4_ microresonator with SIL	[54]/2019
Si_3_N_4_ microring resonator	FWM	1.6 × 10^7^	15–40 GHz	1540–1570 nm	N.A.	N.A.	Commercial DFB laser butt coupled to microring with SIL; soliton immediately generated by turning on pump laser	[55]/2020
Si_3_N_4_ microring resonator	FWM	2.5 × 10^6^	∼1 THz	1070–2140 nm	−21 dBm (single soliton power @ ∼2 μm)	N.A.	Hybrid integration of InP-based DFB laser coupled to Si_3_N_4_ resonator with SIL	[56]/2021
Si_3_N_4_ microring resonator	FWM	7 × 10^6^	100 GHz	1520–1580 nm	N.A.	∼200–300 Hz (fundamental linewidth)	III–V pump laser heterogeneously integrated on Si through bonding and coupled to microring with SIL	[52]/2021
Si_3_N_4_ microring resonator	FWM	>1.0 × 10^7^	30 and 35 GHz	1520–1570 nm (30-dB width = 36.5 nm)	1.4 mW (soliton power)	N.A.	DFB laser butt coupled to a Si_3_N_4_ resonator with SIL	[57]/2021
Si_3_N_4_ racetrack resonator	FWM	>2.6 × 10^8^	43.2 GHz	∼1554–1558 nm	> −10 dBm (for 10 comb teeth)	A few Hz up to 40 Hz (fundamental linewidth)	DFB laser butt coupled to a Si_3_N_4_ racetrack resonator with SIL	[58]/2021

In 2018, Pavlov et al. [77] reported the transformation of an ordinary FP laser diode to an ultra-narrow linewidth light source through SIL by coupling to a whispering gallery mode (WGM) microresonator. The WGM microresonator acts as both external cavity for linewidth narrowing and nonlinear cavity for Kerr comb generation. The linewidth narrowing is contributed by the surface Rayleigh backscattering within the WGM microresonator [77]. The backscattering excites the counter-propagating mode which is feedback to laser cavity. Hence, the laser cavity can be locked to the resonance of the microresonator. More details on the analysis and theory of SIL can be found in ref. [78]. In ref. [77], the soliton Kerr comb generation was also demonstrated with comb linewidth of ∼1 kHz, span of 30 nm and repetition rate of ∼12.5 GHz. Although the light source reported in ref. [77] contains free-space coupling and is not a fully integrated system, the demonstration paves a significant step for compact OFC sources.

An additional point worth mentioning is that the SIL approach discussed earlier brings several advantages for FWM-based soliton comb generation compared with the traditional approach. These advantages include simplified structure (without the need for optical isolation) [56], lowered power consumption (without the need for thermal tuning) [54], reduced comb signal linewidth [52], and enhanced comb stability [52, 57]. In the meanwhile, one disadvantage of SIL approach is reduced dispersive wave power [56]. More details on these advantages and disadvantages are covered in later discussions in the current section.

**Figure 2:**
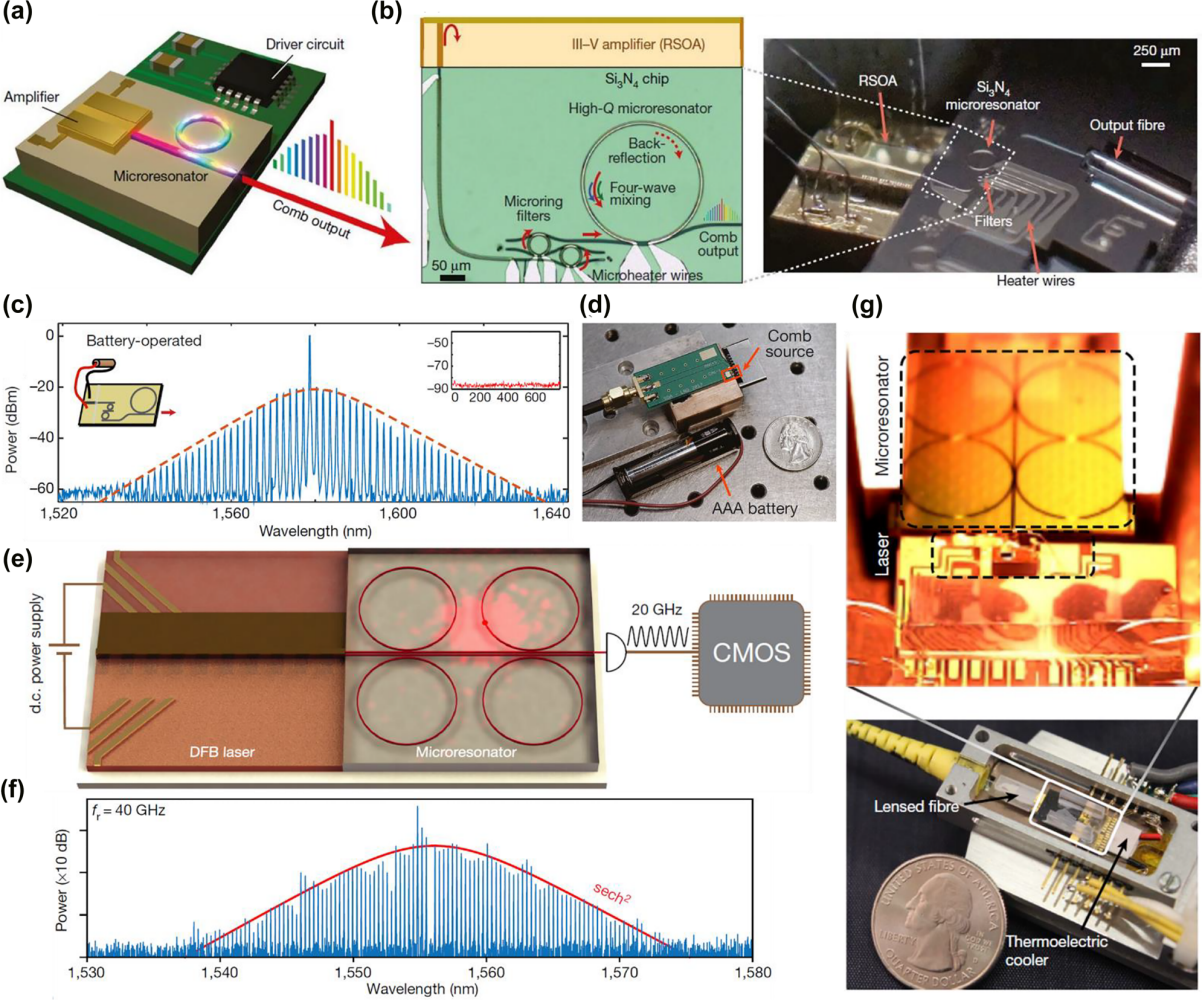
Hybrid integrated electrically driven OFC generated through FWM in high-*Q* resonator. (a) Schematic of integrated OFC generator pumped by an on-chip amplifier. (b) Left panel: microscopic image of RSOA edge coupled to Si_3_N_4_ laser cavity. Two microring filters form the Vernier structure. High-*Q* microring resonator acts as reflective mirror as well as comb line generator. All are integrated with thermal heater. Right panel: Photo of assembled device. (c) Soliton spectrum from OFC powered by AAA battery. Left inset: schematic of OFC powered by battery. Right inset: RF spectrum of OFC showing low-noise state. *Y* axis: power in dBm; *X* axis: RF frequency in MHz. (d) Photo of integrated comb source (highlighted in red) on PCB together with the battery and a US quarter coin (for scaling purpose). (a)–(d) are adapted with permission from Springer Nature, Nature [53]. Battery-operated integrated frequency comb generator, by Stern et al. Copyright 2018. (e) Schematic of a turnkey soliton OFC formed by a high-*Q* Si_3_N_4_ microring resonator integrated with a commercial DFB pump laser driven by a DC power supply. (f) Single-soliton spectrum with repetition rate of 40 GHz fitted by sech^2^ curve in red. (g) Photo of microresonator chip integrated with pump laser packaged in a butterfly box as a compact system. (e)–(g) are adapted with permission from Springer Nature, Nature [55]. Integrated turnkey soliton microcombs, by Shen et al. Copyright 2020.

In the same year, Stern et al. [53] demonstrated a battery powered OFC source, with III–V gain medium integrated with the microring cavity. The conceptual schematic of the integrated OFC generator is shown in Figure 2(a). A reflective semiconductor optical amplifier (RSOA), which is commercially available (Thorlabs SAF1126), is used as the III–V gain medium for the pump source. It is edge coupled to Si_3_N_4_ laser cavity, as illustrated by the images in Figure 2(b). The high-*Q* microring resonator acts as both reflective mirror to provide feedback through Rayleigh scattering and OFC generator to produce comb lines through FWM. The soliton spectrum from OFC powered by AAA battery is captured as shown in Figure 2(c). The spectrum with single-soliton profile spans from 1530 to 1630 nm. A photo of integrated comb source (highlighted in red) on printed circuit board (PCB) together with the battery and a US quarter coin (for scaling purpose) are illustrated in Figure 2(d). The integration of III–V amplifier with microring resonator provides a portable and robust solution for OFC generator with ultra-low power consumption [53]. In the following year, Raja et al. [54] reported an electrically pumped soliton OFC by using III–V laser diode butt coupled to Si_3_N_4_ microresonator. The resonator has high intrinsic *Q* value of >1 × 10^7^. The III–V laser diode is self-injection locked to microresonator along with the narrowing of the laser linewidth and formation of the soliton. The authors observed the transition from SIL-based single-mode lasing to Kerr comb and soliton formation in microring resonator by tuning of the laser diode current. The low-noise soliton state was also observed through heterodyne measurement. In comparison with the report discussed earlier [53], the OFC system here does not require thermal tuning and also simplifies the process for soliton generation [54]. The system provides a solution for compact, low power consumption and low-cost OFC generator for applications requiring high volume production [54].

In 2020, Shen et al. [55] reported a turnkey soliton OFC integrated with a pump laser. A commercial distributed feedback (DFB) pump laser driven by a direct current (DC) power supply is butt-coupled to a high-*Q* Si_3_N_4_ microring resonator, with the schematic shown in Figure 2(e). Although four rings are integrated on the same chip, only one ring resonator is selected for further characterizations. The intrinsic *Q* of the microring is measured to be 1.6 × 10^7^. The single-soliton spectrum generated by the resonator with repetition rate of 40 GHz is plotted in Figure 2(f). The microresonator chip integrated with pump laser is packaged into an industry standard butterfly box as a compact system, with photo shown in Figure 2(g). The turn-key operation for soliton comb generation was theoretically described and experimentally demonstrated in ref. [55]. Soliton generation was realized through the combination of laser SIL and nonlinear resonator response. The experimental demonstration of turnkey operation highlights a simplistic method of soliton comb generation without the need for photonics and electronics control circuitry.

**Figure 3:**
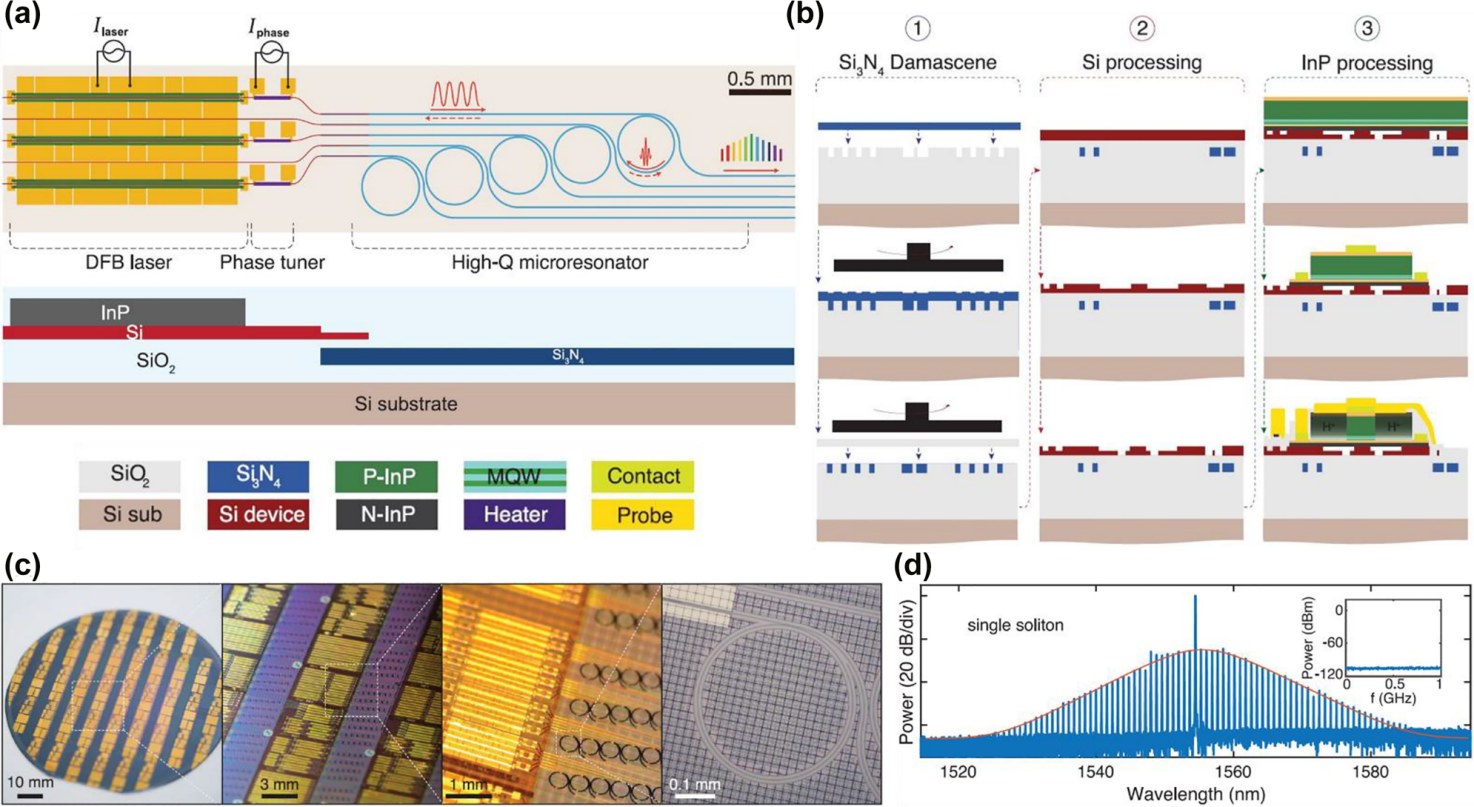
Heterogeneously integrated electrically driven OFC generated through FWM in high-*Q* resonator. (a) Top panel: Schematic of fully integrated OFC generator, including DFB laser as pump, thermo-optic phase tuner for alignment, and high-*Q* ring resonator for comb generation. The backscattered signal (red dotted line) triggers the SIL which assists in soliton formation within the microring resonator. Bottom panel: Cross section of the designed device. (b) Device fabrication process including three steps: (1) Si_3_N_4_ waveguide fabrication process through damascene process followed by a high-temperature annealing (left column); (2) Si process to pattern waveguide structure (middle column); (3) InP process and metal contact formation for pump laser (right column). (c) Photo of fabricated 100-mm wafer and zoom-in microscopic images of the device. (d) Optical spectrum of single-soliton state from device. Inset: RF spectrum of single-soliton state. (a)–(d) are adapted from [52]. Reprinted with permission from AAAS.

A more recent work reported in 2021 by Xiang et al. [52] takes a step further and reports the monolithic integration of indium phosphide (InP) DFB laser with high-*Q* Si_3_N_4_ microring resonator on Si substrate for OFC generation. The schematic of the device is illustrated in Figure 3(a) top panel, including laser as pump source, thermo-optic phase tuner to align pump signal with microring resonant wavelength, and high-*Q* microring resonator for OFC generation. The backscattered signal, as indicated in red dotted line in Figure 3(a) top panel, triggers the SIL which assists the soliton formation within the microring resonator [52]. The coherence of injection locked pump signal can be transferred to the comb lines, which enables narrow comb linewidth of around 200–300 Hz. The cross section of the designed device is illustrated in Figure 3(a) bottom panel. The device fabrication process is illustrated in Figure 3(b). There are three key steps: the first step is the Si_3_N_4_ waveguide fabrication process through damascene process followed by an annealing process, as shown in the left column. The second step is the Si processing to pattern the waveguide structure, as shown in the middle column. The third step is InP processing and metal contact formation, as shown in the right column. The photo of fabricated 100-mm wafer as well as the zoom-in microscopic images of the device are illustrated in Figure 3(c). The optical spectrum of single-soliton state from the fabricated device is plotted in Figure 3(d), with inset showing its RF spectrum. The soliton state can be maintained for hours in lab environment without external control. Such stability is contributed by the monolithic integrated photonics device and the pump-microresonator coupling through SIL [52]. Also, the CMOS-compatible process enables the mass-production of integrated OFC sources, which can be applied in high-capacity transceivers and data centers.

Furthermore, in the same year, Briles et al. [56] reported an octave-spanning OFC by hybrid integration of InP DFB laser and a microresonator using the SIL approach. The SIL-based OFC spans from around 1 μm up to 2 μm wavelength. The octave spanning OFC reported in ref. [56] reveals a key step in self-referenced OFC on integrated photonics platform. Also, in the same work, the SIL approach is compared with other integration strategies for comb generation through simulation. These integration strategies include direct current modulation of optically isolated pump laser as well as ring resonator with photonic crystal structure pumped by optically isolated laser. The simulation analysis reveals the strengths and weaknesses of each integration strategy. For SIL-based comb generation, although it has a simplified structure and enhanced stability, it suffers from reduced dispersive wave power.

Moreover, the report by Voloshin et al. [57] studied the dynamics of soliton formation via pump laser SIL and reported theoretical model of laser SIL to microresonator. Using the theoretical model, the work also compared dynamics for soliton generation between the SIL approach and the conventional approach using pump laser with an optical isolator. Also, a hybrid soliton OFC consisting of DFB laser butt-coupled to a Si_3_N_4_ microring resonator was demonstrated in the same work. The report provides a guideline for future development of fully integrated electrically driven OFC devices implementing laser SIL [57]. In addition, SIL also enables ultra-narrow linewidth (Hz level) of semiconductor laser hybrid integrated with an ultra-high-*Q* racetrack resonator, as recently reported by Jin et al. [58]. With the same configuration, Kerr comb operation was also demonstrated, whose line spacing is suitable for dense wavelength division multiplexed (DWDM) communication system. The demonstration of narrow-linewidth laser and low-noise OFC using CMOS-compatible foundry marks an important step towards mass-manufacturing of coherent optics and photonics systems [58].

## 3 OFC generation through mode locking

In addition to the FWM approach discussed in the earlier section, ML is the other major approach to generate OFC. Semiconductor MLL diode is an ideal OFC generator contributed by the advantages of compactness, robustness, wide gain BW, low cost and low power consumption [59, 79, 80]. ML in a laser can be categorized as the following three types based on mechanisms: active mode locking (AML), passive mode locking (PML), and self-mode locking (SML). AML is achieved by directly adding a modulation signal to the laser cavity, while PML is achieved by implementing a saturable absorber (SA) to modulate the loss within the cavity. Compared to AML, PML is more commonly used, and enables shorter pulses to be generated. Different from AML and PML, SML enables OFC generation without using active or passive modulation. It works based on the spatial hole burning (SHB) effect together with the FWM effect within the laser cavity. Since SML does not use SA within the cavity, it enables higher laser output power in comparison with PML. More details and mechanisms on these three types of ML can be found in ref. [73].

The ML mechanisms mentioned above and key specifications of MLL-based OFC are summarized in Table 2. From Table 2, it can be found that the OFCs based on MLL typically can achieve repetition rate from sub-GHz up to tens of GHz. The wavelength range covers from a few nm up to tens of nm. The laser maximum output power ranges from mW level to tens of mW level. In comparison with the FWM-based OFCs summarized in Table 1, generally, MLL-based OFC can achieve lower repetition rate and higher output power. Ref. [73] also provides detailed key metrics comparison between the MLL-based OFC and FWM-based OFC.

**Table 2:** Summary of fully integrated electrically driven OFC based on MLL.

Materials and	Comb generation	Repetition	Wavelength	Maximum	Comb	Remarks	Reference/
structures	mechanism	rate	range/span	power	linewidth		year
N.A. (semiconductor optical amplifier)	PML	10.16 GHz	*λ*_center_ ∼ 1563 nm; 6.4 nm (3-dB BW); 8.7 nm (10-dB BW)	2 mW	15.41 kHz (RF linewidth)	Monolithically integrated MLL with a MZI to flatten output spectrum	[60]/2016
N.A. (III–V gain)	PML	4.71 GHz	*λ*_center_ ∼ 1580 nm; 6.5 nm (3-dB BW)	6 mW	50 kHz (optical linewidth)	MLL injection locked to a narrow linewidth source to reduce the optical linewidth	[61]/2016
InP-based QW	PML	21.5 GHz	*λ*_center_ ∼ 1535 nm; 14 nm (3-dB BW)	>1 mW	450 kHz (RF 3-dB linewidth)	Wide spectral BW from MLL, with an external semiconductor optical booster amplifier	[62]/2017
InGaAsP-based MQW	PML	1 GHz	*λ*_center_ ∼ 1600 nm; 12 nm (10-dB BW)	∼0.8 mW	<400 kHz (3-dB optical linewidth); 0.9 kHz (10-dB RF linewidth)	III–V-on-Si as optical amplifiers and SA for MLL; low repetition rate enabled by long cavity	[63]/2017
GaAs-based QD	PML	19 GHz	1319–1320 nm	N.A.	N.A.	Wafer-bonded QD MLL laser on Si	[64]/2018
InAs/InGaAs QD	PML	31 GHz	*λ*_center_ ∼ 1316 nm; 1305–1330 nm	>40 mW	100 kHz (3-dB RF linewidth)	First QD passively MLL directly grown on GaP/Si substrate	[65]/2018
AlGaInAs/InP MQW	PML	100 GHz	*λ*_center_ ∼ 1610 nm; 8.05 nm (3-dB BW); 30 nm (20-dB BW)	10.15 mW	N.A.	Comb with 100 GHz line spacing from passively MLL; short pulse duration of 440 fs in QW semiconductor MLL	[59]/2020
InGaAsP/InP QW	PML	50 GHz	*λ*_center_ ∼ 1573 nm; 33 or 34 lines > −3 dBm	90 mW	∼50 kHz (RF 3-dB linewidth)	High-performance MLL as comb and pulse (<500 fs) sources	[66]/2020
InGaAsP/InP QW	SML (SHB; FWM)	23.04, 42.36, GHz (design 1); 30.5 GHz (design 3)	∼ 1590 nm (design 1 & 2); ∼ 1322 nm (design 3)	1 mW	On the order of 100–200 kHz (RF linewidth)	FM comb; simple compact QW-based laser diode with high repetition rate OFC output	[67]/2020
InGaAsP QW	SML (SHB; FWM)	19–25 GHz	*λ*_center_ ∼ 1550 nm; 6 nm (5-dB BW)	1 mW	On the order of 200 kHz (RF linewidth)	FM diode laser OFC can be powered by battery	[68]/2020
GaSb-based QW	SML (SHB; FWM)	∼19.3 GHz	*λ*_center_ ∼ 2060 nm; 20 nm (40-dB BW)	50 mW	∼700 kHz (optical comb linewidth)	FM OFC around 2060 nm wavelength from single-section GaSb-based QW diode laser	[69]/2020
InP coupled with Si_3_N_4_	AML, PML	1.19 GHz	1553.5–1554 nm	1 mW (0 dBm in fiber)	N.A.	Both AML and PML are demonstrated on integrated MLL	[81]/2020
InP-based QW coupled with Si_3_N_4_	AML, PML	360 MHz	*λ*_center_ ∼ 1551 nm; 2.8 nm (30-dB BW)	N.A.	N.A.	Low repetition rate MLL based on AML and PML	[82]/2021
InAlGaAs QW	SML (SHB; FWM)	∼19.4 GHz	1683–1685 nm (under 290 mA)	8 mW	∼1 kHz (RF linewidth)	FM OFC in 1.65 μm wavelength band	[70]/2022

In 2016, the study by Corral et al. [60] demonstrated an OFC generator based on a monolithically integrated MLL fabricated from a multi-project wafer (MPW) in a foundry. The laser is integrated with a Mach–Zehnder interferometer (MZI) to flatten the generated comb spectrum. A follow-up work by the same group [62] reported a monolithically integrated MLL-based OFC with wide spectral BW (14 nm 3-dB BW) also fabricated from a MPW in a foundry. A semiconductor optical amplifier is also integrated on the same chip to boost the output power. In the same year, the study by Uvin et al. [61] reported the linewidth reduction of OFC source using a MLL injection locked to a narrow linewidth source to reduce the optical linewidth of the comb signal. The original linewidth of MLL was in MHz level. After reduction it reached 50 kHz, which is the linewidth of the source used in injection locking. The reduced linewidth enables narrower channel bandwidth and therefore increased spectral efficiency in a communication system [61].

**Figure 4:**
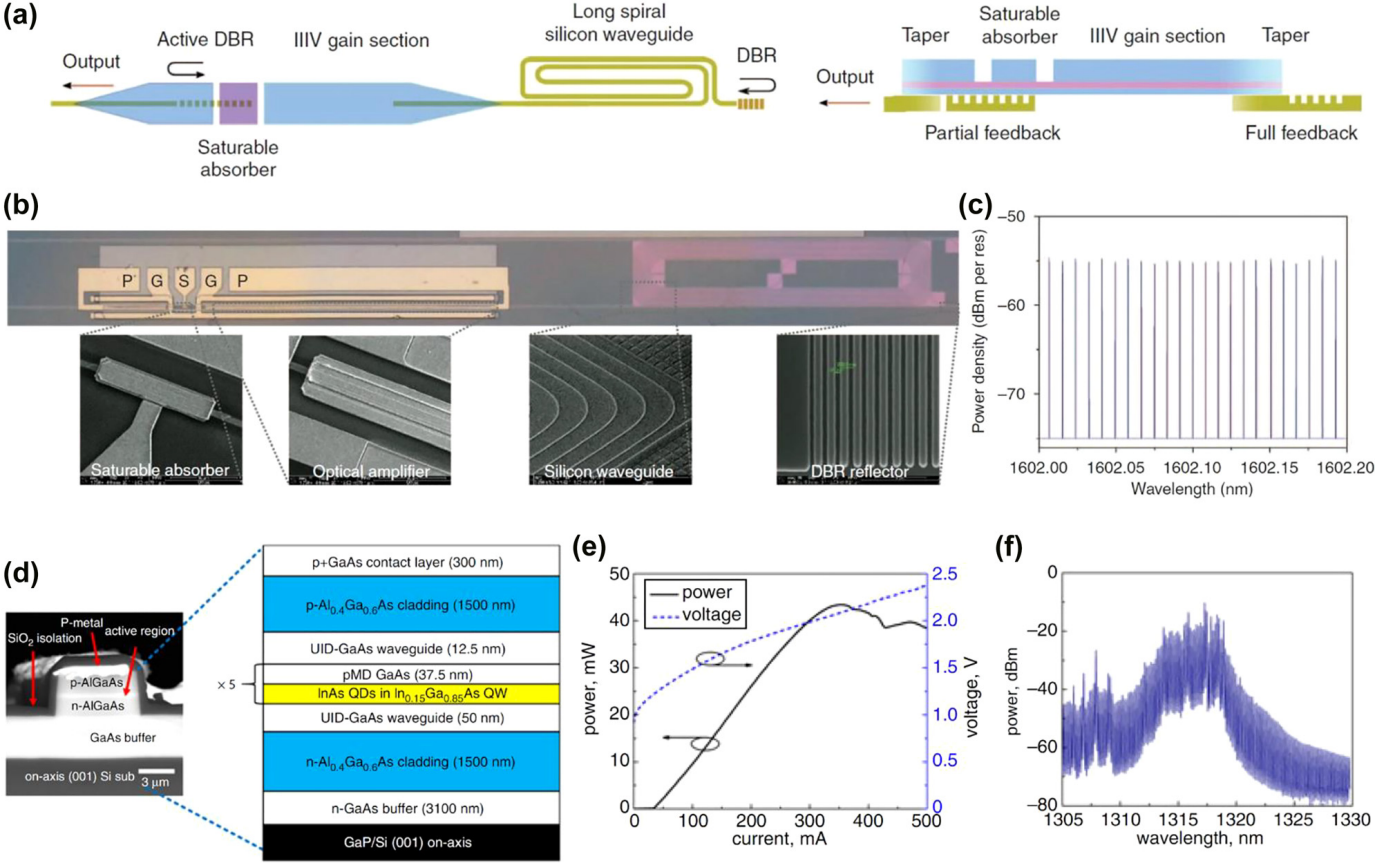
Fully integrated electrically driven OFC based on passively MLL. (a) Schematic (left) and cross-section (right) of the MLL. (b) Microscopic image of the laser and SEM image of each component. (c) A portion of the equally spaced comb lines. (a)–(c) are adapted with permission from [63]. Licensed under a Creative Commons Attribution 4.0 International License. (d) SEM image of epitaxially grown QD laser on Si (left) and cross-section schematic with layer details (right). (e) Output power and voltage with respect to bias current at 20 °C. (f) MLL emission spectrum driving at 470 mA bias current. (d)–(f) are adapted with permission from [65]. © 2018 IEEE.

In the following year, Wang et al. [63] demonstrated an OFC using InGaAsP-based multi-quantum well (MQW) epitaxial stacks as gain material and SA. They are heterogeneously integrated through adhesive die-to-wafer bonding. The schematic and cross section of the MLL are illustrated in Figure 4(a) left and right panel, respectively. The MLL cavity consists of III–V active gain sections, SA, distributed Bragg reflector (DBR) grating mirrors, and long spiral Si waveguide. The microscopic image and the SEM image of each component are also shown in Figure 4(b). A record low repetition rate of 1 GHz is reported, contributed by the long cavity length of low-loss Si waveguide. The dense comb line (>1400 equally spaced comb lines) is promising for high-resolution spectroscopy. A portion of the equally spaced comb lines captured by high-resolution optical spectrum analyzer is illustrated in Figure 4(c).

In 2018, the work by Davenport et al. [80] reported experimental results for optimization of different components in a MLL heterogeneously integrated on Si through bonding process. Also, by using bonding approach for integration, Kurczveil et al. [64] demonstrated quantum dot (QD) comb laser enabled by PML. Besides employing bonding as the integration approach, Liu et al. [65] reported the first QD passively MLL directly grown on GaP/Si substrate. The SEM image of epitaxially grown QD laser on Si and the cross-section schematic with layer details are illustrated in Figure 4(d). The laser has a deep-etched ridge waveguide structure. Its fabrication process is CMOS compatible, and the laser has the potential to be applied in large-scale Si photonics platform in the future [65]. The MLL output power and voltage with respect to bias current at 20 °C is plotted in Figure 4(e). The maximum output power of 40 mW and threshold current of 34 mA can be observed. At 470 mA bias current, the emission spectrum of the MLL is captured as shown in Figure 4(f).

More recently, in 2020, Hou et al. [59] demonstrated OFC with comb line spacing of 100 GHz and ultra-short pulse duration of 440 fs by using asymmetric MQW-based passively MLL. Asymmetric MQW MLL has QW with thickness variation for broadband emission. It demonstrated 3-dB BW of 8.05 nm, and 20-dB BW of 30 nm. Such high BW makes the laser suitable as a comb source in a communication system [59]. In the same year, Zander et al. [66] reported high-performance comb lasers with more than 33 comb lines and 50 GHz line spacing, which enable optical transmission of >1 Tb/s. Furthermore, remarkable output power of the laser was reported as around 90 mW in ref. [66]. In addition, the performance of QD and QW lasers are compared in the same work. QD MLL has better comb spectrum uniformity and lower relative intensity noise. In the meanwhile, QW laser is found to have lower optical linewidth and higher internal quantum efficiency. Both are promising for high data rate (>1 Tb/s) optical communication systems.

**Figure 5:**
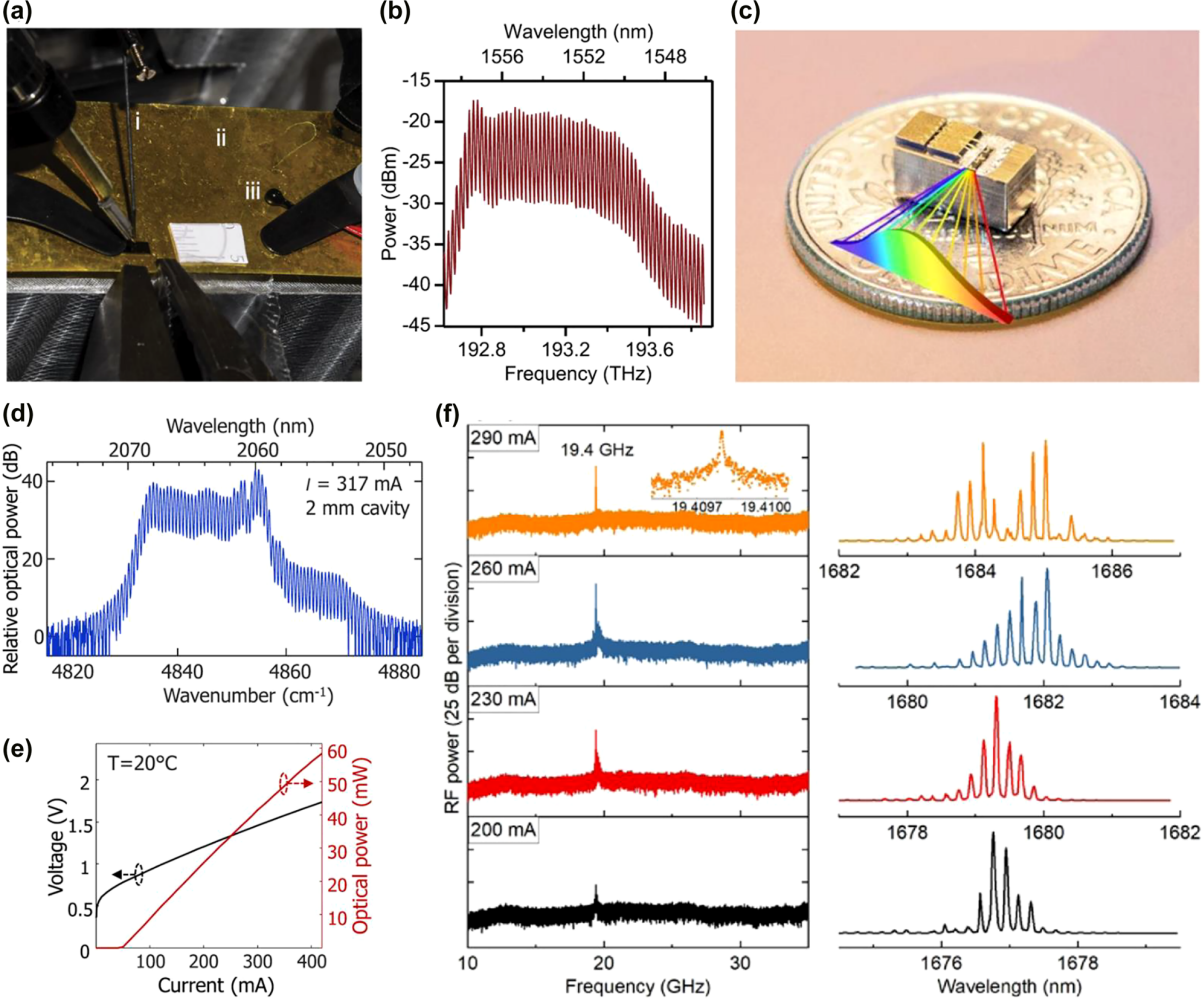
Fully integrated electrically driven OFC based on self MLL. (a) Photograph of the chip containing 21 laser diodes mounted on testing stage for OFC generation. (i) a tungsten probe, (ii) return electrode, (iii) thermistor. A 5 mm ruler is included for scaling purpose. (b) A representative laser diode emission spectrum. (a)–(b) are adapted with the permission from [68]. Licensed under a Creative Commons Attribution 4.0 International License. (c) Photograph of QW laser based OFC generator wire-bonded on a gold-plated copper-tungsten submount. (d) OFC spectrum from the laser with cavity length of 2 mm, under driving current of 317 mA, and working around 2.06 μm wavelength. (e) Optical power and voltage with respect to bias current of the laser diode working at room temperature. (c)–(e) are adapted with the permission from [69]. Licensed under a Creative Commons Attribution 4.0 International License. (f) RF spectra (left column) and optical spectra (right column) from a FM OFC laser working at 1.65 μm wavelength band. The spectra are captured at four different bias currents (200, 230, 260, and 290 mA) above the lasing threshold. (f) is adapted with the permission from [70] © The Optical Society.

In addition to the works implementing PML approach for comb generation as discussed above, MLLs based on SML approach have also been reported recently. In 2020, Dong et al. [67] demonstrated simple OFCs based on frequency modulated (FM) semiconductor QW lasers. In comparison with the above mentioned pulsed MLLs, OFC generation using FM approach has several advantages including getting rid of the carrier-induced phase effects inside cavity, avoiding the use of a SA and hence contributing to ease of design and manufacturing [67]. The OFC is generated through the SHB and FWM effects within the laser cavity. Three laser designs have been demonstrated, with two designs working around 1.5 μm wavelength, and one design working around 1.3 μm. Also, one of the designs with asymmetric QW gain structure has superior performance in terms of BW. More comb specifications are summarized in Table 2. In the same year, a follow-up work by Day et al. [68] demonstrated a battery-operated OFC generator based on diode laser by using the same approach. A photograph of the chip mounted on testing stage is illustrated in Figure 5(a). The chip has a typical dimension of 1–2 mm in length and 2–4 mm in width, containing 21 laser diodes. The photograph includes (i) a tungsten probe, (ii) return electrode, (iii) thermistor. It also includes a 5 mm ruler for scaling purpose. A representative laser diode emission spectrum is captured as shown in Figure 5(b). The emission spectrum of laser diode powered by AA battery has also been captured and reported in ref. [68]. Also, dual-comb spectroscopy of molecular gas has been demonstrated using two devices from a single chip. These demonstrations show the potential of the integrated OFC generators [68].

2-μm wavelength regime is an interesting spectrum regime as a new communication wavelength window with various photonics devices demonstrated [83], [84], [85], [86], [87], [88], [89], [90], [91], [92]. The work by Sterczewski et al. [69] demonstrated a FM OFC operating around 2.06 μm wavelength using GaSb-based QW laser diode. The photograph of QW laser wire-bonded on sub-mount is shown in Figure 5(c). The generated OFC spectrum from a 2-mm long FP cavity under driving current of 317 mA is plotted in Figure 5(d). It shows that most of the power is within 10-nm-wide part of the spectrum, with 35 comb lines and line spacing of 19.3 GHz. At room temperature, the optical power and voltage with respect to bias current is plotted in Figure 5(e). The optical output power of the self-starting comb can reach 50 mW, with <1 W electrical power consumption. The average power per comb line can be estimated to be >1 mW.

More recently, in 2022, the study by Li et al. [70] demonstrated a FM OFC working at 1.65 μm wavelength band generated by a single-section QW laser. The chip-scale OFC in the wavelength band of 1.65 μm has the potential application in methane gas sensing as well as communications [70]. The RF spectra and optical spectra of the QW laser under four different bias currents above the lasing threshold are plotted in Figure 5(f) left and right column, respectively. At 200 mA, the beat signal in RF is still weak, and the laser is in chaotic regime [70]. As the driving current is increased to 230 mA, the laser is in FM mode locking regime, with clear RF beat signal at 19.4 GHz. The RF signal-to-noise ratio reaches maximum at 260 mA, and drops a bit when current is further increased to 290 mA. In the optical spectrum domain, the OFC at 1.65 μm wavelength can be clearly observed under these driving currents.

Besides the MLLs based on PML and SML discussed above, MLLs based on AML approach have also been reported recently [81, 82]. The structure of MLLs is based on InP optical amplifier butt coupled with Si_3_N_4_ chip. The Si_3_N_4_ waveguide has low propagation loss of a few dB/m, and hence enables long laser cavities to achieve low repetition rates. Low repetition rates of 1.19 and 0.36 GHz have been reported using laser cavity length of 7.7 and 26.31 cm, respectively.

An additional note worth mentioning is that at mid-infrared wavelength regime, there is also significant progress on comb generation based on quantum cascade laser using mechanisms including ML and Kerr nonlinearity. Further details can be found from the recent publications [93], [94], [95], [96]. Mid-infrared OFC is an attractive area in the scientific research community as well as for practical applications since a lot of molecules have their fingerprint in this wavelength regime.

## 4 Summary and outlook

To sum up, in this review, firstly, the background and motivation for the development of fully integrated electrically driven OFC generators for communications are introduced. Followed by that, the recent progress on the fully integrated OFC generator based on FWM in high-*Q* resonators, and OFC generator based on MLL are reviewed and summarized in Sections 2 and 3 respectively. For each section, the focus is on the recent demonstrations in the past five years, with key specifications of OFC summarized in a table for comparison.

Further development of fully integrated electrically driven OFC generator can be directed in the following ways. Firstly, OFC based on second order nonlinear effect can be further explored. OFC generated through second order nonlinear effect (e.g., Pockels effect) has been demonstrated with higher optical conversion efficiency and lower power threshold compared with Kerr comb, which is contributed by the higher *χ* ^(2)^ susceptibility compared with *χ* ^(3)^ susceptibility [97]. Besides the advantage of higher efficiency, the comb spectrum covers both fundamental and harmonic frequencies contributed by the cascaded second-harmonic/sum-frequency generation and optical parametric oscillation [97]. In the meanwhile, the demonstration so far employs an off-chip pump source. The comb generation mechanism based on second order nonlinear effect can be better understood, and challenges in pump source integration with second order nonlinear photonics platform need to be overcome. Fully integrated electrically driven OFC using second order nonlinear effect is expected to achieve higher power efficiency, lower power threshold and wider spectrum coverage.

Secondly, fully integrated OFC generator based on electro-optic (EO) effect can be further developed. Compared with the comb generation through FWM or MLL, the OFC generation based on EO effect has the advantages of flexibility in frequency spacing and central wavelength [98]. The EO-based comb generation has been demonstrated on different photonics platforms including Si [98, 99] and lithium niobate (LN) [100], [101], [102], with external laser source coupled onto the chip. The EO comb on LN reported in ref. [100] demonstrated a remarkably broad wavelength coverage, spanning the entire L-band for telecommunication. More recently, the integration of laser source on LN photonics platform has been demonstrated [103], which shows promise for the development of fully integrated electrically driven EO comb with superior performance.

Thirdly, the exploration of novel nonlinear optical materials on CMOS-compatible photonics platform, such as Si-rich SiN [104], and scandium-doped aluminum nitride (AlN) [105, 106] can be conducted. The enhanced nonlinear optical effects of the materials have the potential to achieve higher conversion efficiency on devices. Furthermore, the wide transparency window of AlN and SiN enables comb generation at wavelengths from visible to mid-infrared range [107], [108], [109]. Also, the CMOS-compatibility of these materials enables low-cost wafer-scale fabrication of various functional devices for future mass production [110], [111], [112], [113], [114], [115], [116], [117], [118]. In the meanwhile, there are still challenges in fabrication to overcome in order to make low loss waveguide using Si-rich SiN and scandium-doped AlN. Fabrication processes including etching and annealing can be further optimized to make these materials for practical applications.

Lastly, on-chip pump laser integration on Si is driving the trend for compact, robust, and low-cost OFC generator. Integration approaches including hybrid integration [53, 55] and heterogeneous bonding [52] have been demonstrated for OFC generator based on FWM in high-*Q* microresonator. Direct growth of III–V on Si has also been demonstrated for lasers only [32, 119, 120], but the integration of laser through direct growth approach for fully integrated electrically driven OFC remains to be explored. The challenges in fabrication and integration need to be overcome. In the near future, pump laser sources directly grown on Si wafer is expected for wafer-scale low-cost OFC generator fabrication.

## List of abbreviation

AlN: aluminum nitride
AML: active mode locking
BW: bandwidth
CMOS: complementary metal-oxide-semiconductor
DBR: distributed Bragg reflector
DC: direct current
DFB: distributed feedback
DWDM: dense wavelength division multiplexed
EO: electro-optic
FM: frequency modulated
FWM: four-wave mixing
GaP: gallium phosphide
InP: indium phosphide
LN: lithium niobate
ML: mode locking
MLL: mode-locked laser
MPW: multi-project wafer
MQW: multi-quantum well
MZI: Mach-Zehnder interferometer
OFC: optical frequency comb
PCB: printed circuit board
PML: passive mode locking
QD: quantum dot
QW: quantum well
RF: radio frequency
RSOA: reflective semiconductor optical amplifier
SA: saturable absorber
SEM: scanning electron microscope
SHB: spatial hole burning
Si: silicon
SIL: self-injection locking
SML: self-mode locking
Si_3_N_4_
: silicon nitride
WGM: whispering gallery mode
